# 

**Published:** 1823-10

**Authors:** 


### BOOKS RECEIVED FOR REVIEW.

Leçons sur les Epidemies et l'Hygiene publique. Par F. EM. FODERÈ. Tom. ii.—8vo. Paris, 1823.

Observations on Fractures of the Neck of the Thigh-bone; being an Appendix to the Work on Dislocations and Fractures of the Joints. By Sir ASTLEY COOPER, Bart. F.R.S. Surgeon to the King.—4to. Longman and Co.

### NOTICES TO CORRESPONDENTS.

*We were much surprised to see, in the* John Bull *Newspaper of Sunday the* 14th *instant, a letter from* Sir J. Hippisley *respecting the Tread-Mill, which was sent to us for insertion exclusively, as we believed. On application to our printers, we found that the portion of our Journal containing that letter was printed off; otherwise it would have been omitted, as we are not in the habit of suffering our Correspondence to be anticipated by a newspaper*

